# Risk factors profile and outcomes of stroke in low resources settings: a prospective and multicentric study in a sub-Saharan Africa country

**DOI:** 10.3389/fneur.2026.1903821

**Published:** 2026-09-15

**Authors:** Daniela Sebastião, Evander Lucas, Pedro Lamborne, Antonino Francisco, Mauer Gonçalves, Mário Fresta, Adilson J. M. de Oliveira

**Affiliations:** 1 Faculty of Medicine, Center for Advanced Studies in Medical Education and Training (CEDUMED), Agostinho Neto University, Luanda, Angola; 2 Department of Teaching and Research in Internal Medicine and Dermatology, Faculty of Medicine, Agostinho Neto University, Luanda, Angola; 3 Neuroscience Center, Clínica Girassol, Luanda, Angola

**Keywords:** Angola, neurology, outcomes, risk factors, stroke

## Abstract

**Introduction:**

Stroke is a leading cause of mortality and disability worldwide, with a disproportionate burden in low- and middle-income countries. In sub-Saharan Africa, stroke occurs at younger ages, is characterized by a high proportion of hemorrhagic stroke, and is associated with poorer clinical outcomes. This study aimed to describe the risk factors, stroke subtypes, and clinical outcomes of patients admitted to tertiary hospitals in Luanda, Angola.

**Methods:**

A prospective, observational, multicenter cohort study was conducted including patients diagnosed with ischemic or hemorrhagic stroke who were admitted between May and October 2024 to three tertiary hospitals in Luanda, including two public and one private hospital. Functional outcomes were assessed using the modified Rankin Scale at hospital discharge and at 30, 60, and 90 days. Statistical significance was set at *p* < 0.05.

**Results:**

A total of 165 patients were included, with a mean age of 57.1 ± 13.5 years, and 55.7% of them were younger than 60 years. Males accounted for 52.1% of the sample. Ischemic stroke was the most common subtype, although hemorrhagic stroke represented 46% of cases. Hypertension (84.4%) and sedentary lifestyle (81.8%) were the most prevalent risk factors. At hospital discharge, 18% of patients were functionally independent, whereas 77% had functional dependence. Overall 90-day mortality was 13.3% (22/165), including 4.8% (8/165) in-hospital mortality and 8.5% (14/165) post-discharge mortality during follow-up.

**Conclusion:**

In this cohort, stroke occurred at younger ages and was associated with a high proportion of hemorrhagic stroke, substantial functional dependence, and significant mortality. Hypertension was the main modifiable risk factor. These findings reinforce the need for effective primary prevention strategies, as well as improved screening and control of hypertension in Angola.

## Introduction

Stroke is the leading cause of acquired disability in adults worldwide and the third leading cause of death globally (1, 2). In 2021, stroke accounted for approximately one in six cardiovascular deaths worldwide (3, 4).

In Africa, studies published over the last decade have reported annual stroke incidence rates of up to 316 per 100,000 population, prevalence rates of up to 1,460 per 100,000 population, and 3-year mortality rates exceeding 80% (3, 4). In addition, stroke frequently affects Africans between the fourth and sixth decades of life (5). The burden of stroke increases substantially with age, accounting for approximately one in every 70 Disability-Adjusted Life Years (DALYs) among individuals aged 15–49 years and nearly one in every 15 DALYs among those aged 50–69 years (6).

Africa bears the highest global burden of hypertension, the most important modifiable risk factor for stroke. Although part of the increasing stroke burden has been attributed to gains in life expectancy across the continent, people of African descent experience stroke at younger ages and have worse clinical outcomes compared with populations in high-income countries (3). In Angola, although epidemiological data remain limited, available studies suggest a particularly severe stroke profile characterized by early onset, a high proportion of hemorrhagic stroke, and unfavorable outcomes (7–9). Data from hospital-based studies in Luanda indicate that stroke predominantly affects younger individuals compared to high-income countries, directly impacting the economically active population (8, 10, 11). In a study conducted at a private tertiary hospital in Luanda, hemorrhagic stroke accounted for 44% of cases despite the predominance of ischemic stroke, while hypertension was present in nearly two-thirds of patients (7).

More alarming findings have been reported among younger populations. In patients younger than 46 years admitted with hypertensive emergency to a public hospital in Luanda, more than 80% presented with hemorrhagic stroke as the initial manifestation, and in-hospital mortality exceeded 30% (12). These findings highlight not only the severity of stroke in this setting but also major deficiencies in hypertension detection and control (8, 13).

The magnitude of hemorrhagic stroke becomes even more evident in national cohort studies involving predominantly patients younger than 60 years, high rates of hypertension, and elevated mortality rates (8, 9, 14). In three tertiary hospitals in Luanda, overall mortality exceeded 50%, while functional independence was observed in fewer than one-third of survivors (8). Hematoma volume was a major determinant of prognosis, with particularly high mortality among patients with large hematomas, emphasizing both the clinical severity of the disease and the therapeutic limitations faced in the local context (14).

More recent studies have reinforced this concerning epidemiological profile by demonstrating substantial delays in seeking medical care, a higher frequency of hemorrhagic stroke among younger individuals, and a direct association between elevated blood pressure levels and worse clinical outcomes (9–11). In addition, the high prevalence of modifiable risk factors—including hypertension, alcohol consumption, obesity, sedentary lifestyle, diabetes mellitus, and dyslipidemia—highlights a widespread cardiovascular risk profile within the community (8, 15).

However, prospective multicenter data on stroke characteristics, risk factors, and clinical outcomes in Angola remain scarce. Therefore, this study aimed to describe the demographic characteristics, vascular risk factors, stroke subtypes, and clinical outcomes of patients admitted to tertiary hospitals in Luanda, Angola.

## Methods

### Study design

The authors present a prospective, observational, multicenter study of stroke patients conducted across three tertiary hospitals.

The objective of this study was to describe the demographic characteristics, vascular risk factors, stroke subtypes, and clinical outcomes of patients admitted to tertiary hospitals in Luanda, Angola.

### Setting and dates

This cohort study consecutively enrolled patients admitted either to the Neurology Ward or to the Intensive Care Unit (ICU), according to clinical severity and the need for intensive care, at three tertiary hospitals in Luanda, Angola (Josina Machel Hospital, Prenda Hospital, and Girassol Clinic) between May and October 2024. All eligible patients were recruited consecutively and were followed for a minimum of 30 days, with outcome assessment extending to 90 days. The patient enrolment flowchart is presented in Figure 1.

**Figure 1:**
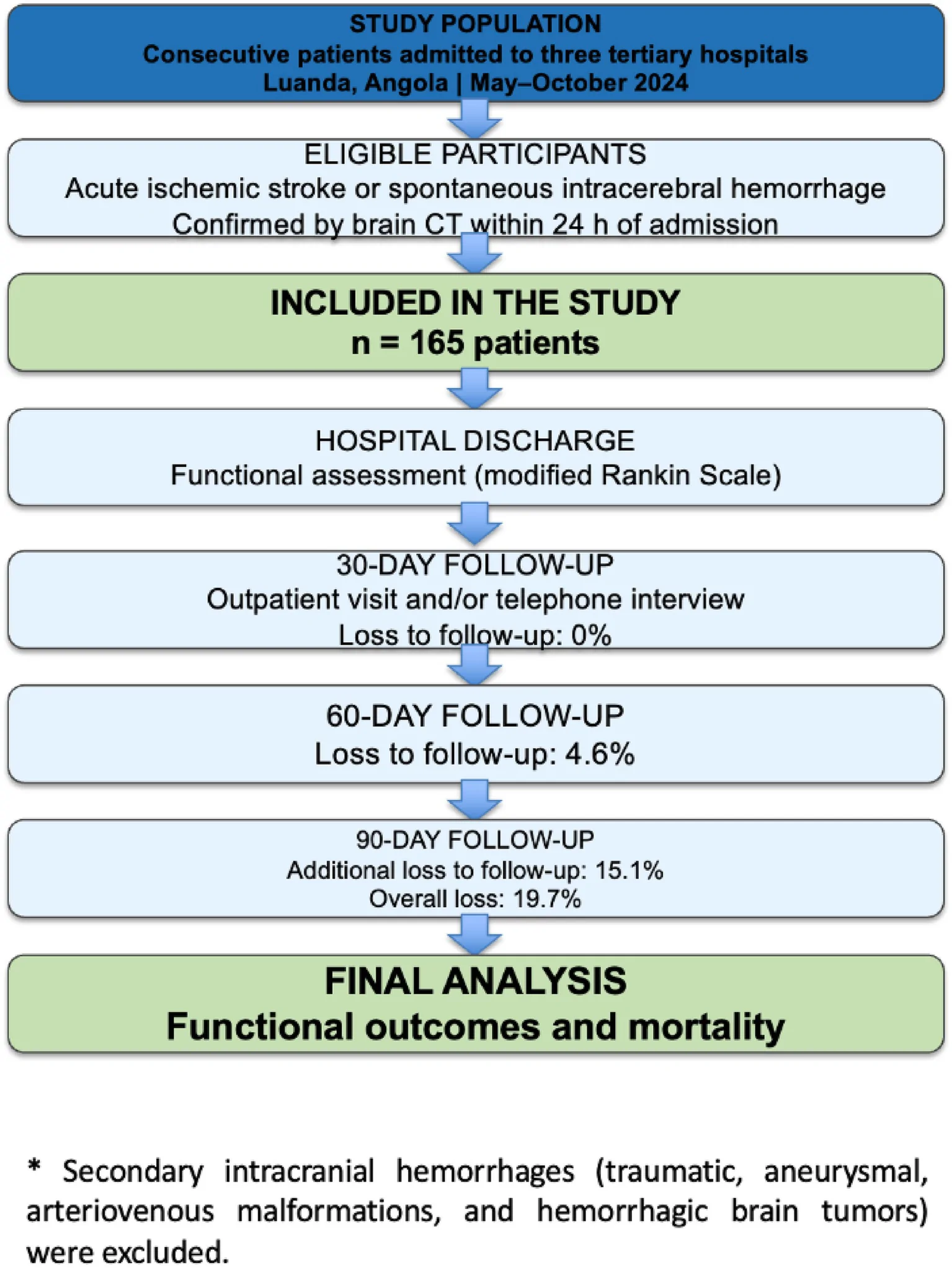
Patient enrollment and follow-up flow diagram.

### Inclusion criteria

1. Admission to one of the participating hospitals during the study period;
2. Age ≥18 years;
3. A diagnosis of acute ischemic stroke or spontaneous intracerebral hemorrhage confirmed by brain computed tomography (CT) performed within the first 24 h after hospital admission.
4. Provision of written informed consent by the patient or a legal representative.

### Exclusion criteria

1. Non-spontaneous intracranial hemorrhage (ICH);
2. Patients in whom the diagnosis of ischemic or hemorrhagic stroke is not confirmed within 24 h.
3. Patients who do not provide informed consent to participate in the study.
4. Pediatric patients.

### Variables

1. Demographic variables: age and sex.
2. Clinical variables: stroke subtype (ischemic or hemorrhagic), vascular risk factors (hypertension, diabetes mellitus, dyslipidemia, smoking, alcohol consumption, obesity, previous stroke, and sedentary lifestyle).
3. Ischemic stroke etiology according to the Trial of Org 10,172 in Acute Stroke Treatment (TOAST) classification (16).
4. Neurological severity was assessed using the National Institutes of Health Stroke Scale (NIHSS) at hospital admission and discharge (17). NIHSS data were missing for 21 patients at admission and 30 patients at discharge. Therefore, the paired analysis was restricted to patients with complete NIHSS assessments at both time points, and no imputation of missing values was performed.
5. NIHSS scores were expressed as median and interquartile range (IQR), and admission and discharge scores were compared using the Wilcoxon signed-rank test.
6. Radiological variables: brain CT findings (stroke type and location);

### Procedures

Data were collected using structured case report forms adapted from the INTERSTROKE study methodology (8). Clinical interviews were conducted with patients and/or relatives or caregivers familiar with the patients’ medical history and lifestyle habits. The forms were used to collect sociodemographic information and data regarding vascular risk factors.

Vascular risk factors were assessed according to standard clinical practice. Information regarding hypertension, diabetes mellitus, smoking, alcohol consumption, family history of stroke, and other medical conditions was obtained through structured interviews with patients or, when necessary, their relatives or caregivers, and was confirmed by review of the available medical records whenever possible. Risk factors identified by physical examination, such as obesity, were assessed at hospital admission according to standard clinical criteria routinely applied in neurological practice.

All vascular and behavioral risk factors were assessed according to standard clinical definitions routinely used in neurological practice, and no study-specific operational definitions were adopted. Sedentary lifestyle was determined from the patient’s (or caregiver’s) self-reported level of regular physical activity during the structured interview, following routine clinical assessment.

The diagnosis of stroke was based on the acute clinical presentation and confirmed by a brain CT scan performed within the first 24 h after hospital admission. Patients were classified as having ischemic stroke or spontaneous intracerebral hemorrhage according to the clinical and neuroimaging findings.

Neuroimaging examinations were initially interpreted by the on-duty radiologist at each participating hospital and were subsequently independently reviewed by a senior investigator with expertise in neuroradiology. In cases of disagreement regarding stroke subtype or lesion characteristics, the images were reviewed by a third neuroradiologist, and the final classification was established by consensus.

The etiological work-up included review of the clinical history, physical and neurological examination, routine laboratory investigations, electrocardiography, transthoracic echocardiography, carotid Doppler ultrasonography, computed tomography angiography, Holter monitoring, and brain magnetic resonance imaging, whenever clinically indicated and available. Laboratory investigations included, when available, blood glucose, glycated hemoglobin, lipid profile, renal function, and coagulation tests.

For intracerebral hemorrhage, diagnosis and anatomical location were determined from the initial CT scan.

Functional outcome was assessed using the modified Rankin Scale (mRS), Brott et al. (17) a 7-point ordinal scale ranging from 0 (no symptoms) to 6 (death). Functional status was evaluated at hospital discharge and at 30, 60, and 90 days after stroke using structured mRS assessments conducted by trained investigators, either in person during outpatient visits or through structured telephone interviews. Assessors were blinded to stroke subtype. For outcome analysis, functional independence was defined as an mRS score of 0–2, functional dependence as an mRS score of 3–5, and death as an mRS score of 6.

Because etiological classification requires integration of clinical, neuroimaging, cardiac, vascular, and laboratory findings, the final TOAST classification was established during hospitalization, after completion of the diagnostic work-up and availability of complementary investigation results. The initial etiological assessment was performed by the attending neurologists and subsequently reviewed by the senior investigators using all available clinical and diagnostic information. Cases in which the available diagnostic information was insufficient to establish a specific mechanism were classified as stroke of undetermined etiology.

### Outcomes

Functional outcome assessed using the modified Rankin Scale (mRS) at hospital discharge and at 30, 60, and 90 days.

Mortality was assessed separately as in-hospital mortality and cumulative 3-month mortality. In-hospital mortality was defined as death occurring during the index hospitalization, whereas cumulative 3-month mortality included all deaths recorded from hospital admission through the 90-day follow-up.

### Statistical analysis

Data were entered into a structured database and analyzed using the Statistical Package for the Social Sciences (IBM SPSS Statistics version 20.0). Data normality was assessed using the Shapiro–Wilk test. Continuous variables were expressed as mean ± standard deviation or median and interquartile range, as appropriate. Categorical variables were expressed as absolute and relative frequencies. Group comparisons were performed using the Mann–Whitney U test for continuous variables and the chi-square test or Fisher’s exact test for categorical variables, as appropriate. Statistical significance was defined as *p* < 0.05, with a 95% confidence interval. No adjustment for multiple comparisons was applied because comparisons of individual risk factors between public and private hospitals were exploratory rather than prespecified confirmatory hypothesis tests. Accordingly, the reported *p*-values should be interpreted as exploratory.

At each follow-up time point, functional outcome analyses were restricted to patients with available outcome data at that assessment, and patients lost to follow-up were excluded from the corresponding denominator. Deaths were retained cumulatively as mRS 6 at subsequent follow-up assessments.

No formal *a priori* sample-size calculation was performed because the study was designed as a consecutive, time-defined hospital-based cohort. All eligible patients presenting to the participating centers during the predefined 6-month recruitment period were prospectively enrolled; therefore, the final sample size was determined by the number of eligible patients presenting during the study period.

### Ethical and administrative procedures

This study was approved by the Scientific and Pedagogical Council of the Centre for Advanced Studies in Medical Education and Training of the Faculty of Medicine, Agostinho Neto University under document number 37/VDACIEPG/FM/2024. The study was conducted after formal authorization from the scientific and administrative boards of the participating hospitals. All participants or their legal representatives provided written informed consent. Participant confidentiality and anonymity were ensured in accordance with the principles of the Declaration of Helsinki.

## Results

A total of 165 patients were included in the study, with a mean age of 57.1 ± 13.5 years. There was a slight male predominance (86 men and 79 women), corresponding to a male-to-female ratio of 1.09:1.

Ischemic stroke was the predominant subtype, accounting for 54% of cases, while hemorrhagic stroke represented 46% (Figure 2).

**Figure 2:**
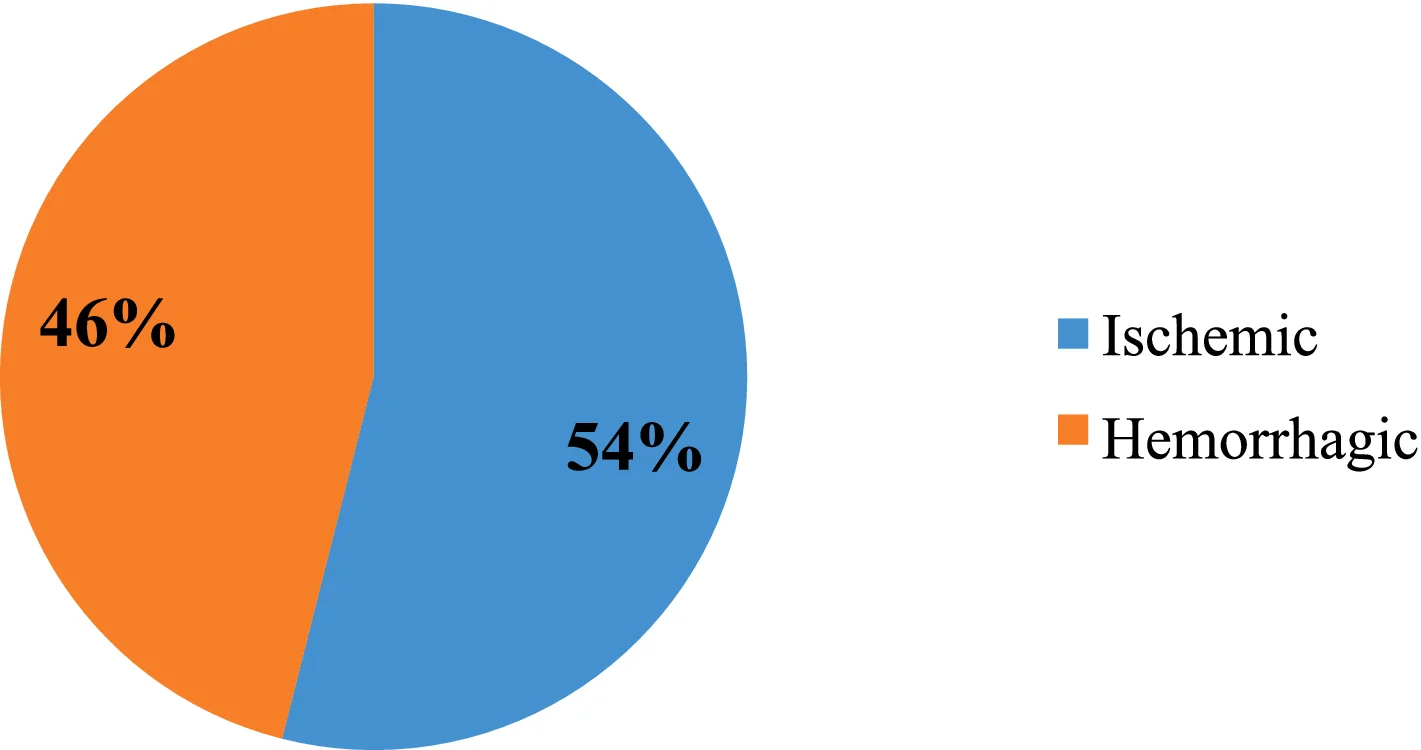
Distribution according to stroke type.

Median NIHSS decreased from 16 at admission to 14 at discharge among patients with complete paired assessments. However, this change was not statistically significant according to the Wilcoxon signed-rank test (*p* = 0.220). Detailed NIHSS results are presented in Table 1.

**Table 1:** NIHSS score at admission and discharge.

Assesment time point	Median	IQR	*N*	Missing
NIHSS admission	16	11–24	144	21
NIHSS discharge	14	8–19	135	30

Wilcoxon signed—rank test 135 Paired observation *P* = 0.220.

According to the TOAST classification, 56% of ischemic strokes were attributed to small vessel disease, whereas 19% had undetermined etiology (Figure 3). Among hemorrhagic strokes, 71.1% of hematomas were located in the gangliocapsular region (Figure 4).

**Figure 3:**
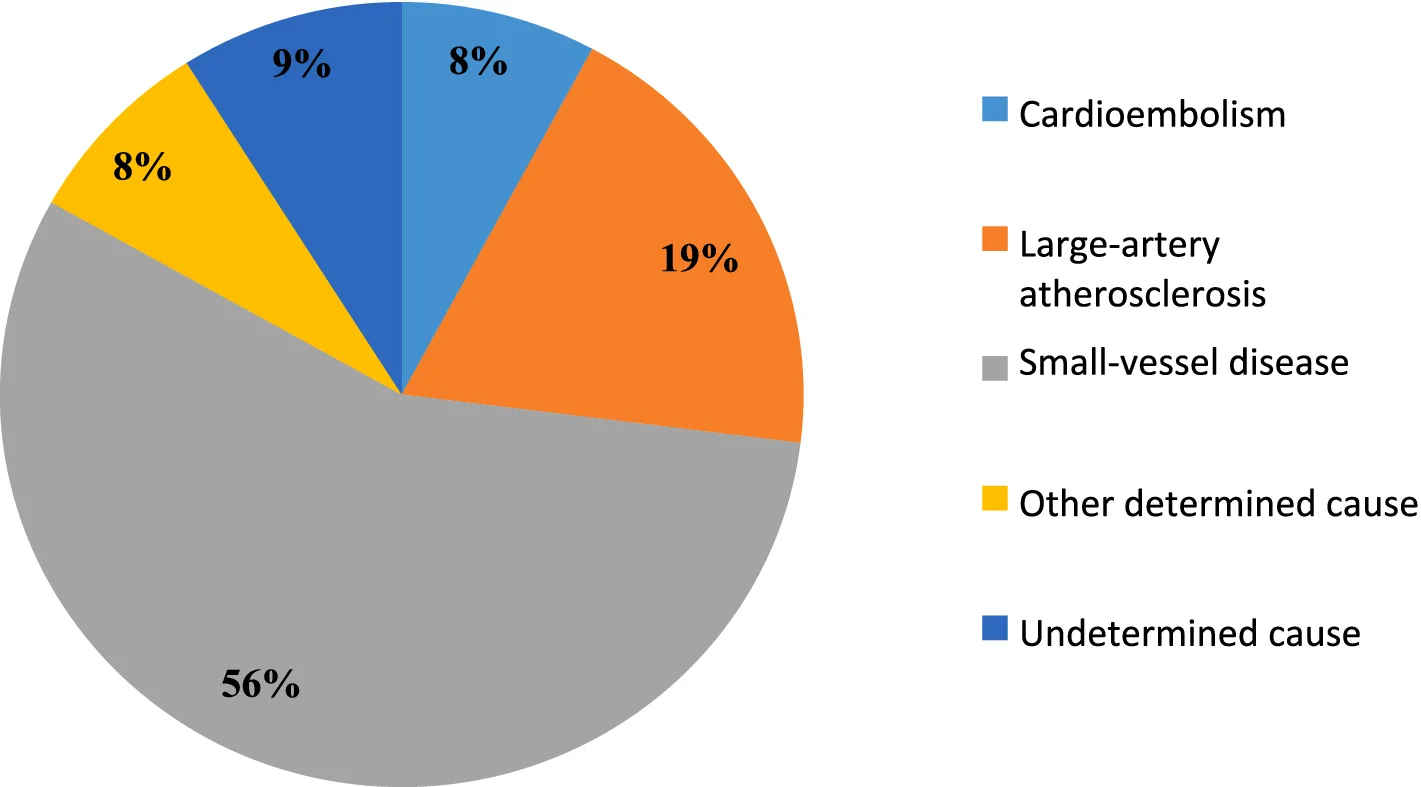
Distribution of ischemic stroke etiologies according to the TOAST classification (*N* = 89). Etiology was determined during the index hospitalization after completion of the available diagnostic work-up (*N* = 89).

**Figure 4:**
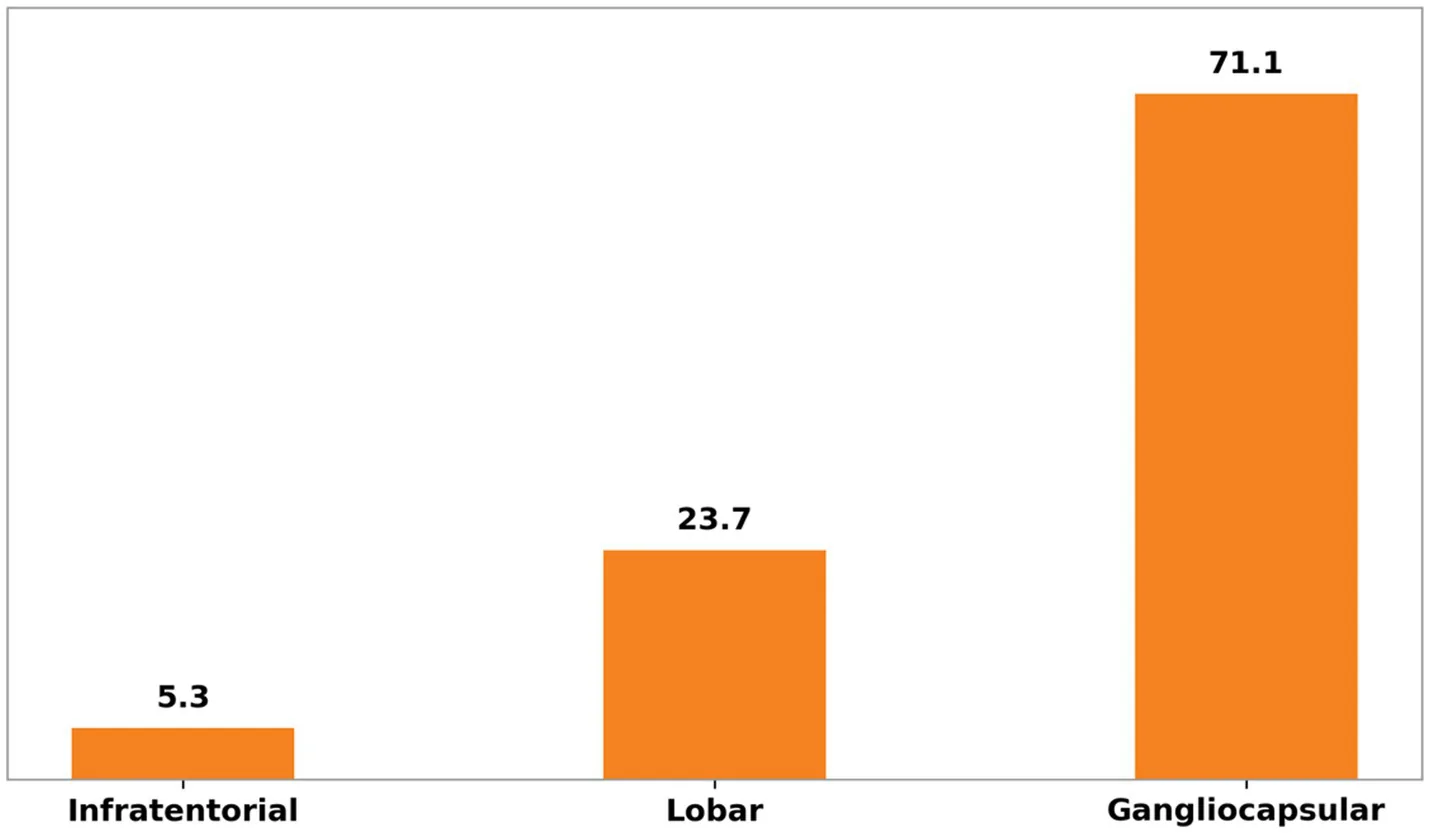
Distribution according to hematoma location in ICH.

In public hospitals, the most prevalent vascular risk factors were sedentary lifestyle (85.2%), hypertension (82.8%), alcohol consumption (50%), previous or family history of stroke (42.6%), obesity (22.1%), smoking (18.9%), and diabetes mellitus (17.2%). In the private hospital, the most frequent risk factors were hypertension (86%), sedentary lifestyle (72.1%), alcohol consumption (46.5%), previous or family history of stroke (46.5%), obesity (41.9%), diabetes mellitus (32.6%), and smoking (9.3%). Diabetes mellitus and obesity were the only risk factors that differed significantly between public and private hospitals (*p* = 0.034 and *p* = 0.024, respectively) (Table 2).

**Table 2:** Distribution according to the frequency of risk factors.

Risk factors	Category	Hospital				Total		*P*
		Public		Private				
		*n*	%	*n*	%	*n*	%	
Hypertension	Yes	101	82.8	37	86.0	138	83.6	0.619
	No	21	17.2	6	14.0	27	16.4	
	Total	122	100	43	100	165	100	
Diabetes	Yes	21	17.2	14	32.6	35	21.2	**0.034**
	No	101	82.8	29	67.4	130	78.8	
	Total	122	100	43	100	165	100	
Alcoholism	Yes	61	50	20	46.5	81	49.1	0.694
	No	61	50	23	53.5	84	50.9	
	Total	122	100	43	100	165	100	
Regular physical activity	Yes	18	14.8	12	27.9	30	18.2	0.054
	No	104	85.2	31	72.1	135	81.8	
	Total	122	100	43	100	165	100	
Family history of stroke	Yes	52	42.6	20	46.5	72	43.6	0.658
	No	70	57.4	23	53.5	93	56.4	
	Total	122	100	43	100	165	100	
Smoking	Yes	23	18.9	4	9.3	27	16.4	0.335
	No	82	67.2	33	76.7	115	69.7	
	Former smoker	17	13.9	6	14.0	23	13.9	
	Total	122	100	43	100	165	100	
Obesity	Yes	27	22.1	18	41.9	45	27.3	**0.024**
	No	60	49.2	19	44.2	79	47.9	
	Undetermined	35	28.7	6	14.0	41	24.8	
	Total	122	100	43	100	165	100	

*p-*values were calculated using Pearson’s chi-square test. Statistical significance was set at *p* < 0.05. Bold values indicates statistical significative diference.

Overall 90-day mortality was 13.3% (22/165), including 7 ischemic and 15 hemorrhagic stroke patients. Eight deaths occurred during the index hospitalization (4.8%; 3 ischemic and 5 hemorrhagic), while 14 deaths occurred after hospital discharge during the 90-day follow-up (8.5%; 4 ischemic and 10 hemorrhagic).

Table 3 summarizes functional outcomes according to stroke subtype. Among patients with ischemic stroke, 19.1% were functionally independent at hospital discharge, whereas 77.5% had functional dependence and 3.4% died during hospitalization. At 90 days, 76.7% of patients were functionally independent, while 23.3% remained functionally dependent, with no deaths recorded during this period.

**Table 3:** Distribution of patient outcomes according to stroke type.

Modified rankin scale for patients with ischemic stroke	Discharge		30 days		60 days		90 days	
	*n*	%	*n*	%	*n*	%	*n*	%
Asymptomatic (0)	0	0	3	3.5	8	10.4	20	33.3
Symptomatic, but without disability (1)	3	3.4	7	8.1	15	19.5	10	16.7
Mild disability (2)	14	15.7	25	29.1	19	24.7	16	26.7
Moderate disability (3)	43	48.3	27	31.4	24	31.2	9	15
Moderately severe disability(4)	24	27.0	21	24.4	9	11.7	5	8.3
Severe disability (5)	2	2.2	1	1.2	0	0	0	0
Death (6)	3	3.4	5	5.8	7	9.1	7	11.7
Total	89	100	86	100	77	100	60	100

Modified rankin scale for patients with hemorrhagic stroke	Discharge		30 days		60 days		90 days	
	*n*	%	*n*	%	*n*	%	*n*	%
Asymptomatic (0)	0	0	1	1.4	10	14.9	13	22.4
Symptomatic, but without disability (1)	3	3.9	7	9.9	10	14.9	12	20.7
Mild disability (2)	10	13.2	18	25.4	11	16.4	5	8.6
Moderate disability (3)	28	36.8	22	31	22	32.8	23	39.7
Moderately disability severe (4)	24	31.6	16	22.5	9	13.4	2	3.4
Severe disability (5)	6	7.9	3	4.2	2	3	0	0
Death (6)	5	6.6	9	12.7	12	17.9	15	25.9%
Total	76	100	71	100	67	100	58	100

Deaths (mRS 6) are reported cumulatively at each follow-up time point. Denominators for functional outcome analyses include patients with available outcome data at each assessment and therefore vary according to loss to follow-up.

Among patients with hemorrhagic stroke, 17.1% were functionally independent at discharge, 76.3% had functional dependence, and 6.6% died during hospitalization. At 90 days, 51.7% of patients achieved functional independence, whereas 43.1% remained functionally dependent and 5.2% died.

Overall, functional outcomes improved progressively over time. At hospital discharge, 18% of patients were functionally independent, while 77% had functional dependence. At 30 days, functional independence increased to 38.9%, reaching 51% at 60 days and 64.4% at 90 days, as shown in Figure 5. The mean hospital length of stay was 10 days.

**Figure 5:**
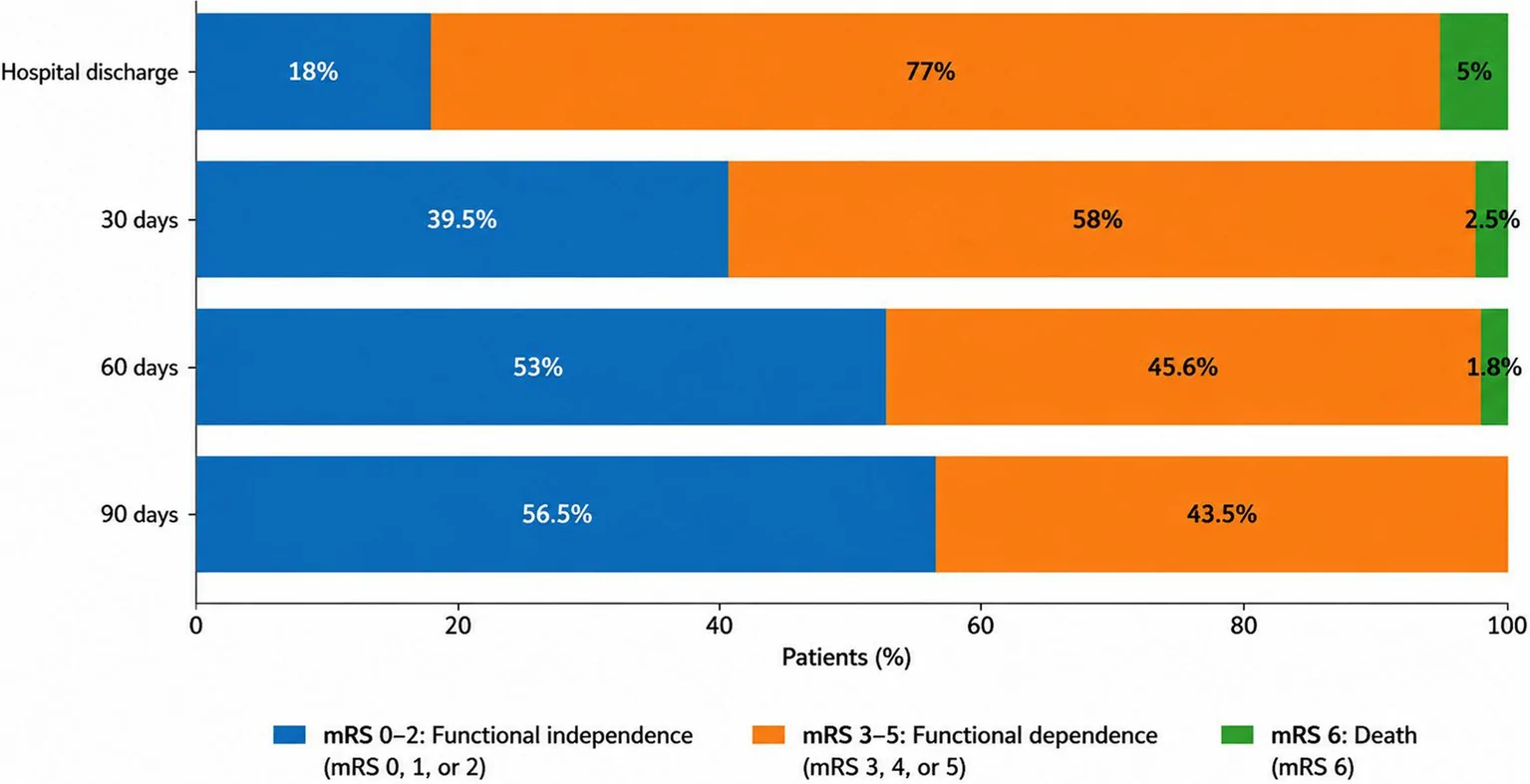
Distribution of stroke patients according to discharge, 30, 60, and 90-day outcomes.

In-hospital mortality was 4.8% (8/165), while cumulative 3-month mortality was 13.3%. Most deaths occurred during hospitalization and within the first month after stroke (Figure 4).

The overall loss to follow-up rate was 19.7%, with no losses between hospital discharge and 30 days, a 4.6% loss between 30 and 60 days, and a 15.1% loss between 60 and 90 days. Complete outcome data were available for 157 patients at 30 days, 144 at 60 days, and 118 at 90 days. There was no loss to follow-up between hospital discharge and 30 days. Loss to follow-up occurred during the subsequent intervals, with a 4.6% loss between 30 and 60 days and a 15.1% loss between 60 and 90 days.

## Discussion

### Socio-demographic characteristics

Our study revealed a particularly young stroke population, with 55% of participants being younger than 60 years. This finding contrasts with the epidemiological profile reported in high-income countries, where stroke predominantly affects older individuals and stroke among younger adults represents a substantially smaller proportion of cases. Studies from high-income countries have reported that approximately 5–20% of strokes occur in younger individuals, although the age cut-off used to define young stroke varies across studies (4). This finding is consistent with data from several sub-Saharan African cohorts and previously published Angolan studies demonstrating earlier stroke onset in African populations (6–9). This pattern likely reflects both the demographic structure of African populations, characterized by a younger age distribution, and the earlier onset and suboptimal long-term control of vascular risk factors, particularly hypertension.

Limited access to preventive healthcare services, low rates of cardiovascular screening, delayed diagnosis, poor adherence to antihypertensive therapy, and socioeconomic barriers may further contribute to the earlier development of cerebrovascular disease in African populations. The public health implications of this profile are substantial, as stroke occurring in younger individuals disproportionately affects economically active and reproductive-age populations, thereby increasing disability, productivity loss, and long-term socioeconomic burden.

### Stroke subtype

Ischemic stroke was the most frequent subtype in our cohort, consistent with global epidemiological trends. However, one of the most striking findings of our study was the exceptionally high proportion of hemorrhagic stroke, accounting for nearly 46% of all stroke cases. This proportion is considerably higher than international estimates, where hemorrhagic stroke accounts for approximately 20% of all strokes, but is similar to findings reported in several sub-Saharan African populations (7–9, 17).

Importantly, the relationship between hypertension and intracerebral hemorrhage should be interpreted according to hemorrhage location. In our cohort, 71.1% of intracerebral hemorrhages were located in the gangliocapsular region. Deep intracerebral hemorrhages are strongly associated with hypertensive small-vessel arteriopathy, whereas lobar hemorrhages may reflect other underlying mechanisms and should not be directly attributed to hypertension. Therefore, although the high prevalence of hypertension in our population may contribute to the substantial burden of ICH, this association is particularly relevant to the predominance of deep hemorrhages observed in our cohort (10, 17).

This finding should also be considered together with the etiological distribution of ischemic stroke. Small-vessel disease accounted for 56% of ischemic strokes in our cohort. Deep ICH and small-vessel disease-related ischemic stroke represent different clinical expressions of cerebral small-vessel disease, for which hypertension is a major risk factor. Thus, rather than considering the high proportion of hemorrhagic stroke in isolation, our findings suggest a broader and disproportionate burden of small-vessel cerebrovascular disease in this population. This pattern may reflect prolonged exposure to inadequately detected or controlled hypertension and may partly explain both the high frequency of deep ICH and the predominance of small-vessel disease among ischemic strokes (10, 14, 17).

Similar findings have been consistently reported in previous Angolan and African studies, reinforcing the central role of uncontrolled hypertension in severe cerebrovascular disease across the region (13, 14). In addition, the high burden of hemorrhagic stroke may place considerable pressure on healthcare systems, particularly in low-resource settings where access to neurocritical care, stroke units, neurosurgical services, and rehabilitation programs remains limited (17, 18).

Approximately 19% of ischemic strokes were classified as having undetermined etiology. This is an established category within the TOAST classification and may occur even after a complete diagnostic work-up when no definite cause can be identified or when multiple potential causes coexist. In our setting, the variable availability of diagnostic investigations, particularly in public hospitals with resource and logistical constraints, may have additionally contributed to the proportion of cases classified as undetermined.

### Risk factors

Because this was a pragmatic multicenter study conducted in a low-resource setting, access to complementary diagnostic investigations was not uniform across the participating hospitals. Therefore, not all patients underwent the same etiological work-up, and investigations were performed according to clinical indication and local availability.

Hypertension was the most prevalent vascular risk factor in our cohort and its importance should be interpreted together with the predominance of deep intracerebral hemorrhage and small-vessel disease among ischemic strokes, both of which are strongly associated with hypertension-related small-vessel cerebrovascular disease. Previous African and Angolan studies have similarly identified hypertension as the most prevalent and clinically relevant modifiable risk factor for stroke (8, 13, 14). The high burden of hypertension in African populations has been associated with rapid urbanization, dietary changes, increased salt intake, sedentary lifestyle, obesity, limited access to healthcare, low disease awareness, and poor long-term treatment adherence (14, 17, 19, 20). Diabetes mellitus and obesity were the only risk factors that differed significantly between public and private hospitals (*p* = 0.034 and *p* = 0.024, respectively). Although other vascular risk factors identified in our study—including diabetes mellitus, smoking, alcohol consumption, obesity, and dyslipidemia—were also frequent, their relative contribution may vary according to local epidemiological characteristics and access to diagnostic resources.

### Outcomes

Regarding clinical outcomes, in-hospital mortality was 4.8%, while cumulative mortality reached 13.3% at 90 days. These figures should be interpreted within the context of the substantial mortality burden reported for stroke in Africa. Akinyemi et al. reported 30-day case-fatality rates ranging from 16.2 to 46% in hospital-based African studies and a pooled 1-month case-fatality estimate of approximately 24% from studies conducted in sub-Saharan Africa (5). These figures are higher than the mortality observed in our cohort, although direct comparisons should be made cautiously because of differences in study populations, stroke subtype distribution, stroke severity, healthcare settings, and duration and completeness of follow-up. Previous Angolan studies have also demonstrated a substantial mortality burden after stroke, particularly among patients with intracerebral hemorrhage (8, 13).

Despite the relatively low in-hospital mortality observed in our cohort, functional disability remained markedly elevated at hospital discharge, with a large proportion of patients presenting significant impairment according to the modified Rankin Scale. Several factors may explain this observation. The high proportion of hemorrhagic stroke and the substantial burden of small-vessel cerebrovascular disease may have contributed to the neurological severity and functional dependence observed in the early phase after stroke (21–24).

Limited access to structured rehabilitation services may also have played an important role. In many low-resource settings, rehabilitation infrastructure remains insufficient, with restricted availability of physiotherapy, occupational therapy, speech therapy, and long-term multidisciplinary follow-up. Delays in rehabilitation initiation and poor continuity of care may further compromise long-term functional recovery (5).

Nevertheless, our findings demonstrated progressive functional improvement over the 90-day follow-up period, suggesting that a substantial proportion of survivors may recover functional independence despite significant disability at hospital discharge. However, the 19.7% loss to follow-up at 90 days should be considered when interpreting these findings, as patients lost to follow-up may have had different functional outcomes from those who completed follow-up.

Taken together, these findings reinforce the need for improved primary and secondary prevention strategies, expansion of acute stroke care infrastructure, and development of accessible rehabilitation programs. Such measures may contribute not only to reducing stroke-related mortality but also to minimizing the substantial burden of long-term disability in Angola and other Sub-Saharan African settings (5).

## Strengths and limitations

This study has several strengths. Its prospective and multicenter design allowed consecutive enrollment of patients across three tertiary hospitals in Luanda, while longitudinal assessment of functional outcomes up to 90 days provided information beyond the acute hospitalization. In addition, the inclusion of both ischemic and hemorrhagic stroke enabled characterization of a broad spectrum of hospitalized stroke patients in this setting.

This study has some limitations that should be acknowledged.

First, the etiological work-up was not uniform across all participants because access to vascular imaging, cardiac investigations, magnetic resonance imaging, and selected laboratory tests varied according to availability at each participating hospital. This may have contributed to the proportion of ischemic strokes classified as having an undetermined etiology.

The loss to follow-up rate of 19.7% at 90 days represents an important limitation of this study. Because functional outcomes were available only for patients who completed follow-up, attrition may have introduced selection bias if patients lost to follow-up had systematically different outcomes from those who remained in the study. Consequently, the observed proportion of functional independence at 90 days may not fully represent the outcomes of the entire cohort and should be interpreted with caution.

Dietary evaluation was limited by the difficulty of adapting the INTERSTROKE dietary risk score to the local context, while lipid profile assessment was restricted by the limited availability of laboratory testing in some participating hospitals.

Because this was a hospital-based study conducted in three tertiary referral centers in Luanda, the cohort may preferentially represent patients with access to tertiary hospital care and may not capture the full spectrum of stroke occurring in the community. Therefore, although the participating institutions are major referral centers, these findings should not be considered population-based estimates or be fully generalized to the entire Angolan population.

Finally, NIHSS assessments were incomplete for a proportion of patients, reducing the sample available for paired analysis of neurological severity between admission and discharge.

## Conclusion

In this cohort, hemorrhagic stroke accounted for a substantially higher proportion of cases than that typically reported in high-income countries, where ischemic stroke predominates. This finding suggests a distinct epidemiological profile in the Angolan population, likely associated with inadequate hypertension control and limited access to healthcare services. Despite progressive functional improvement during the 90-day follow-up period, initial functional dependence and mortality remained elevated. The substantial burden of mortality and residual disability after stroke highlights the importance of preventing both first and recurrent events, particularly through the control of modifiable vascular risk factors. These findings reinforce the need for effective primary and secondary prevention strategies focused on early detection and rigorous control of hypertension and other modifiable risk factors. However, as this was a hospital-based study rather than a population-based study, the findings should not be considered fully representative of the Angolan population.

## Data Availability

The raw data supporting the conclusions of this article will be made available by the authors, without undue reservation.
